# Bioabsorbable Poly(vinyl alcohol)–Citric Acid Dressings: Wound Healing Studies in an Experimental In Vivo Model

**DOI:** 10.3390/ebj6020018

**Published:** 2025-04-08

**Authors:** Jonalba Mendes Pereira, Emilia Angela Lo Schiavo Arisawa, Antônio Luiz Martins Maia Filho, José Figueredo-Silva, Nicoly Alves, Carolina Hahn da Silveira, Lucia Vieira

**Affiliations:** 1 Instituto de Pesquisa e Desenvolvimento, Universidade do Vale do Paraíba, Avenida Shishima Hifumi, 2911, Urbanova, São José dos Campos 12244-000, SP, Brazil; girassol_21@hotmail.com (J.M.P.); nicolynunes.br@gmail.com (N.A.); cahdsilveira@gmail.com (C.H.d.S.); 2 Biotechnology and Biodiversity Research Center, State University of Piaui, R. Lucídio Freitas, 2791-Matinha, Teresina 64003-120, PI, Brazil; almmaiaf@ccs.uespi.br (A.L.M.M.F.); figueredo_silva@hotmail.com (J.F.-S.)

**Keywords:** dressings, bioabsorbable, wound healing, polyvinyl alcohol–citric acid (PVA-AC)

## Abstract

Background: The wound healing process presents notable challenges for nursing teams, requiring extensive knowledge of wound care materials. A nanoparticle-free, bioabsorbable pol-yvinyl alcohol (PVA) with citric acid (CA) dressing produced by simple electrospin-ning was evaluated to treat acute wound healing in rats. This PVA-CA combination promotes crosslinking, increases the dressing capacity of absorption and confers heal-ing properties due to the citric acid antioxidant action. Methods: The dressing was tested in a quantitative experiment on 1.9 cm acute dermatological lesions in rats (*n* = 12), com-paring the PVA-CA-treated group with the untreated control group (CG). Samples were collected at 3, 7 and 14 days after lesion induction to evaluate the inflammatory process and tissue healing. Results: The macroscopic and histological data on the third day showed similar characteristics in both groups; however, after fourteen days, the PVA-CA group exhibited complete healing, accompanied by recomposition of the skin layers, whereas the wounds in the CG did not close completely. Conclusions: The results highlight that electrospun PVA-AC dressings improve healing outcomes and constitute a prom-ising and affordable solution, providing a suitable environment for tissue repair, re-ducing inflammatory cell infiltration, blood vessel formation, and restoration of epi-thelial tissue, reducing the time of the healing process of acute wounds.

## 1. Introduction

Skin integrity is essential in protecting the body from infections and other external agents; when compromised, vulnerability increases, potentially leading to pain and a higher risk of complications [1]. The wound healing process presents notable challenges for nursing teams, requiring a thorough understanding of tissue repair mechanisms, extensive knowledge of wound care materials, and the ability to make informed decisions when selecting the most appropriate dressings. The substantial costs associated with wound care are frequently linked to prolonged hospitalizations and the engagement of multidisciplinary teams to manage potential complications effectively. The high costs associated with wound care are often attributed to extended hospital stays and the involvement of interdisciplinary teams to address possible complications [2]. Selecting the proper dressing is crucial to applying best practices and technologies in patient care, promoting faster and more efficient recovery [2]. This includes dressings designed to maintain moisture, relieve pain, prevent infection, and ensure ease of application and removal [2]. Investing in research and innovation in this field is essential to enhance the quality of care, optimize resource utilization, and reduce the financial strain on healthcare systems. Developing dressings that speed up tissue repair is a growing and vital area in healthcare.

In recent years, biocompatible gels for wound care have emerged as a specialized field that requires technical knowledge and a holistic approach, enabling health professionals to develop systematic and autonomous practices. A variety of biomodulation therapies have been developed to accelerate healing, including photobiomodulation [3], ozone therapy [4], nonthermal plasma [5,6,7], and bio-printed dressings [8]. The effectiveness of these treatments depends directly on the expertise of professionals, who must be appropriately trained to adopt these technologies effectively and safely [4]. Among bio-printed dressings, electrospinning, which produces ultra-fine polymer fibers, has shown promising results [9]. By enhancing treatment effectiveness, electrospinning also supports the autonomy and specialization of healthcare professionals [10,11]. These fibers, with diameters ranging from nanometers to micrometers, have diverse applications, from filtration to tissue engineering [12,13,14]. 

Electrospinning was developed by Rayleigh and Zeleny (1897, 1914) and patented by Formhals (1934); this technique gained recognition only in the 1990s with increased interest in nanotechnology and new materials [15]. Electrospinning offers advantages such as low-cost equipment, reduced reagent use, simple manufacturing, and large-scale production capacity [12]. In tissue engineering, this technique produces nanostructured biomaterials that form a three-dimensional network, mimicking the fibrillar structure of the extracellular matrix and promoting the growth and recovery of damaged tissue, suitable for application on skin wounds [13]. Electrospinning produces nanometer- or micrometer-scale diameter fibers by moving a fluid through an electric field. These fibers can act directly in the healing process by simulating the structural, functional, and chemical properties of the extracellular matrix of biological tissues, aiding in cell adhesion, proliferation, differentiation, migration, and shape regulation [14]. Alternatively, their structure allows nanofibers to function as delivery systems for various substances incorporated into their polymer matrix [14]. Polyvinyl alcohol, PVA, is often used in electrospinning nonwoven materials due to its unique properties. PVA’s high mesh transparency allows for excellent fluid absorption and efficient nutrient and waste exchange for cells [8].

PVA-based hydrogels exhibit elasticity similar to biological tissues, ensuring adhesion to the body, as highlighted by Chouhan et al. (2019) and Dawei Jin et al. (2022), respectively [15,16]. Another critical aspect of PVA is its biocompatibility, which, due to its surface tension, provides good elasticity and mechanical properties. It also offers vapor transmission properties, creating an ideal moist microenvironment for epithelialization [8,17]. Citric acid (CA) is an option for crosslinking agents in a PVA solution, forming covalent bonds between the polymer chains, which increases the mechanical strength and stability of the dressing [18].

The inclusion of citric acid as a crosslinking agent is critical for improving the properties of chitosan/PVA systems. Chang et al. (2025) explored citrate-crosslinked hydrogels prepared via freeze–thaw cycles, achieving improved mechanical strength, water absorption, and thermal stability through optimal crosslinking [19]. Similarly, Pratinthong et al. (2024) demonstrated that incorporating citric acid into carboxymethyl cellulose/PVA hydrogels enhances their drug release capabilities and anti-inflammatory effectiveness, making them ideal for wound healing applications [20]. The crosslinking significantly influences the hydrogel properties, such as porosity, swelling behavior, and biodegradability. Studies indicate that higher crosslinking densities enhance mechanical strength and reduce solubility, whereas lower crosslinking promotes faster degradation and absorption, aligning with specific biomedical needs. These findings underscore the adaptability of chitosan/PVA hydrogels for tailored applications. Using citric acid as a biocompatible crosslinking agent improves the structural and functional properties of chitosan-/PVA-based materials. These advancements facilitate applications in wound healing and drug delivery, as evidenced by the studies from Zahra et al., Chang et al. (2025), and Pratinthong et al. (2024), respectively [18,19,20].

This study evaluated the efficacy of a bioabsorbable dressing produced through simple electrospinning using polyvinyl alcohol (PVA) and citric acid (CA) in promoting acute wound healing in rats. Notably, the dressing was nanoparticle-free, emphasizing a straightforward and biocompatible approach.

## 2. Materials and Methods

This study was an experimental, controlled study with random allocation, and it was interventional with a quantitative approach.

### 2.1. Ethical Considerations

This study was reviewed and approved by the Animal Ethics Committee (CEUA) of the State University of Piauí under protocol number 005708/2024-18.

### 2.2. Location

The research was conducted at the Nanotecplasma Laboratory of the Institute for Research and Development (IP&D) at the University of Vale do Paraíba (Univap) in São José dos Campos, SP, where the electrospun dressings were produced. The dressings were then shipped under standard conditions (ensuring sterility and temperature control) to the Biotechnology Center of the State University of Piauí (UESPI) in Teresina, PI, where they were applied to the PVA group at the Biotechnology Center of the State University of Piauí (UESPI) in Teresina, PI.

### 2.3. Preparation of the PVA Solution

The preparation of the PVA-AC solution began with adding 2.5 g of PVA (molecular weight of 104 Mw) to 25 g of distilled water, and the mixture was stirred at 80 °C until fully dissolved. Next, 0.125 g of citric acid was added for crosslinking, followed by another 30 min of stirring. The solution was allowed to cool before being used in the electrospinning process. After preparing the PVA solution, 3 mL was loaded into a 5 mL syringe and mounted on the electrospinning pump.

### 2.4. Electrospinning Nonwoven Fabric Processing

The main components required for nonwoven fabric processing are a high-voltage power supply (15–20 Kv), a reservoir containing the polymer solution (syringe), a metallic needle, and a metallic collector [21]. The following parameters were used: a flow rate of 1 mLh^−1^, a voltage of 15 kV, and a distance of 11.5 cm between the needle tip and the collector. The voltage between the needle and collector created a droplet that deformed and initiated a jet while the solvent evaporated, resulting in fiber deposition onto the collector. Electrostatic repulsive forces drove fiber formation, with instability interactions determining the final fiber morphology. The electrospinning nonwoven fabric was produced in a controlled environment at 21 °C with 30% humidity. After 3 h, the fibers were deposited on the collector, and the nonwoven material was removed and cut into squares of 4 cm^2^ segments.

**Figure 1:**
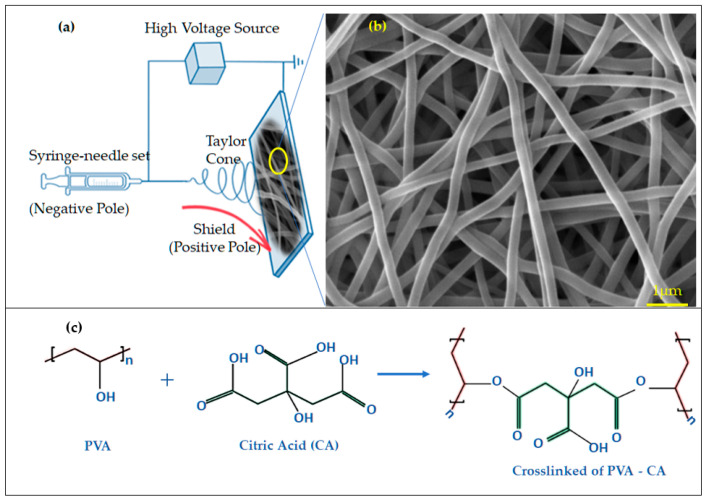
(**a**) Electrospinning system; (**b**) photomicrograph obtained by MEV of PVA fiber morphology; (**c**) crosslinked explanation of PVA + citric acid.

Figure 1a provides a schematic representation of the electrospinning setup using the BioRender software, incorporating free images available within the platform. In contrast, Figure 1b shows an SEM photomicrograph of the resulting PVA fibers, which exhibited uniform morphology, nanometric diameters, and smooth surfaces. The image of the crosslinking process illustrated in Figure 1c is a custom illustration designed by the authors using the BioRender software (2025) using free images, highlighting the role of citric acid (CA) as a crosslinking agent. CA interacts with PVA’s hydroxyl groups (-OH) through its carboxylic acid groups, forming ester bonds during heating [22,23]. This reaction releases water and creates covalent bonds that link the polymer chains [24]. The degree of crosslinking, determined by the concentration of CA, significantly influencing the dressing’s properties, such as mechanical strength, liquid absorption, and biodegradability [21].

Higher CA concentrations result in extensive crosslinking, producing durable dressings with reduced solubility and slower degradation, which is ideal for prolonged wound protection. Conversely, lower CA concentrations yield dressings with faster degradation and higher absorbability, suitable for wounds requiring temporary coverage without frequent dressing changes.

The dressing developed in this study demonstrated a high absorption capacity, a critical feature for cutaneous wound treatment. This reduces the need for manual removal, which could impede healing. Published studies corroborate that tuning the concentration of crosslinking agents in PVA-based dressings enables the customization of key properties, including absorption, degradation, and biocompatibility, essential for effective wound healing [23,24]. Using citric acid as a biocompatible crosslinker enhances the dressing’s functionality without introducing toxicity, further supporting its application in wound care [25].

### 2.5. Dressing Chemical Composition Analyses: Fourier Transform Infrared Spectroscopy (FTIR)-UATR System

Disk-shaped samples of PVA and PVA + CA nonwoven material, approximately 0.5 cm^2^ in size, were analyzed using a PerkinElmer Spectrum 400 FTIR spectrometer equipped with a universal attenuated total reflection (UATR) accessory, United Kingdom. Measurements were conducted following Advancing Standards Transforming Market (ASTM E1252 standards), with 30 scans averaged per spectrum over a wavenumber range of 500–4000 cm^−1^ and a resolution of 4 cm^−1^ [24]. Baseline and UATR corrections were applied using the Savitzky–Golay method (5 points) in the Spectrum software. The percentage of transmittance bands was quantified by calculating the integrated areas of the spectra, processed with the Origin^®^ 2018 software. The degree of chemical crosslinking in PVA following citric acid (CA) incorporation was evaluated by observing changes in specific transmittance bands.

### 2.6. Methodology

Twelve rats (*Rattus norvegicus albinus—Wistar*), male, 60 days old, weighing 250 ± 6.6 g, were kept under the following standard conditions: 25 °C, 60% humidity, 12/12 h light/dark cycle, with free access to food and water. The animals were kept under these conditions for five days before the beginning of the experimental procedures. The animals were divided into two groups (*n* = 6 each): the control group (CG—without treatment) and the PVA group (treated with PVA dressing). Thirty-six wound slice samples were excised and analyzed, eighteen from the control group and eighteen from the PVA group, at experimental times of 0, 3, 7, and 14 days, considering the evolution of the wound healing process phases, i.e., inflammation, proliferation, and remodeling [23].

### 2.7. Experimental Protocol

**Figure 2:**
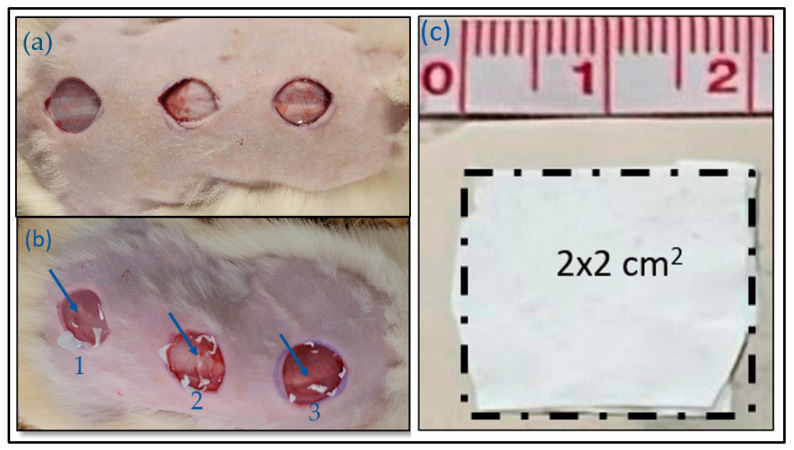
(**a**) Three induced experimental lesions, with approximately a 1.9 cm diameter, immediately after the experimental procedures in the control group; (**b**) PVA group treated with bioabsorbable poly(vinyl alcohol)–citric acid dressings; (**c**) 4 cm^2^ of the PVA dressing.

The experimental protocol began with a surgical procedure based on that of Otterço et al. (2018) [19]. After weighing, the animals were given an intraperitoneal injection of ketamine (60–80 mg/kg) and xylazine (10 mg/kg), followed by dorsal digital trichotomy in a ventral position. Three lesions were created in each animal using a dermatological punch (1.9 cm in diameter) positioned just below the scapula. The skin was then removed with a scalpel, providing an area larger than 2 cm^2^ whilst maintaining a spacing of approximately 2 cm between each lesion. This procedure aimed to minimize the number of animals euthanized after the experiment. Thirty-six samples were excised and analyzed, eighteen from the control group and eighteen from the PVA group, at experimental times of 0, 3, 7, and 14 days, considering the evolution of the inflammatory process’s phases, including the acute, proliferative, and remodeling phases (Figure 2a).

The electrospun dressing was cut into a 4 cm^2^ square and subsequently stored in sterile packaging for use after lesion induction. The dressing adhered to the lesion area without requiring overlapping or secondary coverage, as illustrated in Figure 2a–c. The PVA dressing remained adhered throughout the experiment until its complete absorption. At the experimental periods of 3 and 7 days, one of the three wounds was excised for analysis. After injury induction and treatment, the animals were housed individually in polyethylene cages under the same standard conditions. On the 14th day of treatment, the animals were anesthetized for the removal of the third lesion site and subsequently euthanized using an overdose of ketamine and xylazine.

Skin samples were placed in 10% buffered formalin for 24 h. Two to three transverse samples from each fragment were processed histologically in a Lupetec PT 05 TS histotechnical apparatus (São Carlos, SP, Brazil) and embedded in paraffin. Blocks were sectioned with a Lupetec MRP 09 microtome (São Carlos, SP, Brazil) to obtain 5 µm thick slices.

For qualitative histological analysis, sections were stained with hematoxylin and eosin (H&E) and examined under an Olympus CX31 trinocular optical microscope (model YS100, San Jose, CA, USA) equipped with a digital camera (Bell & Howell, EU 16.0 Plus, Durham, NC, USA). For comparison, the histological images were captured at the center of each lesion.

### 2.8. Data Analysis and Treatment

Macroscopic analysis of tissue repair evolution was performed at experimental intervals of 0, 3, 7, and 14 days using images acquired with a Nikon Coolpix AW130 digital camera (Nishi-oi, Tokyo, Japan) in the primary mode (without flash or zoom) fixed on an aluminum support, 20 cm away from the area of interest. The lesion dimensions were measured with a caliper. Digital images were analyzed using the Image J 1.45 software. The statistical analysis was conducted using the JAMOVI software, version 2.3.28 (The Jamovi project, 2022), available at https://www.jamovi.org (accessed on 9 July 2024). A 95% significance level (*p* < 0.05) was applied throughout.

Data normality was assessed with the Shapiro–Wilk test as normal distribution values in parentheses, as shown in Table 1 (p Shapiro–Wilk: W. area = 0.255; %Reduc = 0.137). Additionally, the high *p*-values in this test indicated a normal distribution.

A two-way ANOVA (*p*-value < 0.05) was performed to compare the groups, analyzing interactions between independent categorical and continuous dependent variables. The ANOVA analysis indicated that post hoc pairwise comparisons were conducted using Tukey’s test (*p* < 0.05) to identify specific time points at which intra-subject effects occurred.

## 3. Results

### 3.1. Chemical Composition Analysis of Dressings by FTIR

**Figure 3:**
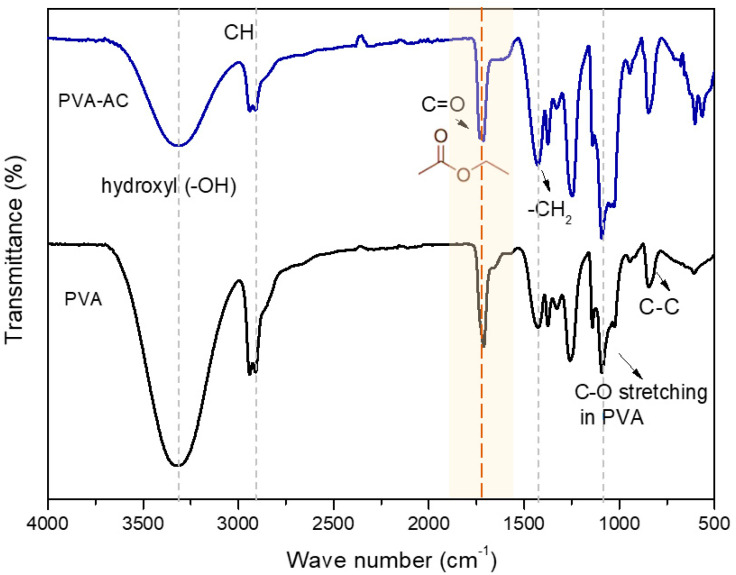
FTIR transmittance plot of the spectra of PVA-CA and of the pure PVA, respectively.

Figure 3 shows an FTIR transmittance plot using the attenuated total reflectance (ATR) method of the spectra of PVA-CA and of the pure PVA presented in the range of 4000–500 cm^−1^, respectively. The characteristic peaks of PVA and its interactions with citric acid (CA) were identified and can be explained as follows: The transmittance bands at 3014–3680 cm^−1^ were attributed to the stretching vibration of the hydroxyl groups (-OH) present in PVA and free non-crosslinked CA molecules. The bands at 2931 and 2853 cm^−1^ were attributed to the asymmetric and symmetrical elongation of the C-H groups, respectively.

The band at 1735 cm^−1^ was attributed to residual carbonyl groups in PVA (from acetate), CH_3_COO, and ester carbonyls formed in the crosslinked hydrogel, highlighted in a yellow transparent bar on the plot. The band at 1426 cm^−1^ corresponds to the in-plane deformation of the -CH_2_- group. A characteristic band between 1000–1180 cm^−1^ was also observed and attributed to PVA’s C-O stretching. Finally, the band at 842 cm^−1^ represents the vibrational stretching of C-C bonds. These bands provide insights into the PVA-CA hydrogel’s molecular interactions and structural modifications.

### 3.2. Macroscopic Analysis of Lesion Area Regression Percentage

**Figure 4:**
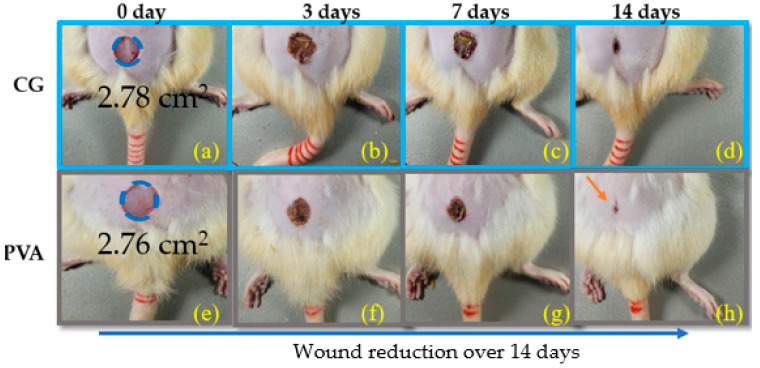
Reduction in wound area of skin lesions over the experimental intervals of 0, 3, 7, and 14 days. The images were acquired with a Nikon Coolpix AW130 digital camera in the primary mode (without flash or zoom) fixed on an aluminum support 20 cm away from the area of interest. Control group (**a**–**d**); PVA-CA (**e**–**h**), respectively.

The macroscopic analysis allowed for calculating the percentage reduction (%Reduc.) in the area of the skin lesions over the experimental periods studied. Images of the lesions were taken at 0, 3, 7, and 14 days to analyze the evolution of the healing process, as shown in Figure 4. Each lesion’s area of the digital images was measured with the Image J 1.45 software. The percentage of regression was determined at each time point by comparing the initial lesion size with measurements taken on the following days (0, 3, 7, and 14 days). The initial area of skin removed using a scalpel was greater than 2 cm^2^. This approach allowed for a quantitative assessment of the healing process, measuring the area of wound closure rates over time in the control and PVA-treated groups. Statistical comparisons between the groups were made to determine the significance of the treatment effects on wound regression percentages, as shown in Table 1.

Table 1 shows the average lesion reduction area. After 14 days, the control group (blue) showed a wound area reduction of 89.7%, while the PVA group (black) exhibited a reduction of 95.9%. The numbers in parentheses indicate the standard deviation.

**Figure 5:**
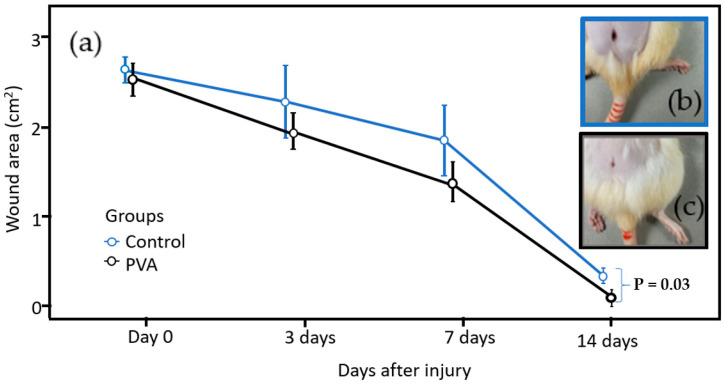
Comparative plot (**a**) of wound healing in the control (**b**) and PVA (**c**) treated groups of albino rats.

Figure 5a compares the wound area reduction between the control and PVA groups. On day zero, the wound areas were similar across both groups, with overlapping error bars on days 0, 3, and 7, indicating no statistically significant difference during these time points.

Over time, the plot shows a more pronounced reduction in wound area in the PVA group compared to the control group. However, no statistically significant difference (*p* < 0.05) was observed during the initial days, and the error bars on days 0, 3, and 7 showed overlap. By day 14, the PVA-treated wounds exhibited near-complete healing, as highlighted by the blue and black square images in Figure 5b,c. The error bars no longer overlapped, and statistics tests indicated a significant difference between the groups (*p* = 0.03).

Close-up views of the wound site in Figure 5c revealed new skin growth in the PVA group, further confirming advanced recovery compared to the control. These findings emphasize the effectiveness of the PVA treatment in promoting wound healing.

### 3.3. Qualitative Histological Analysis

**Figure 6:**
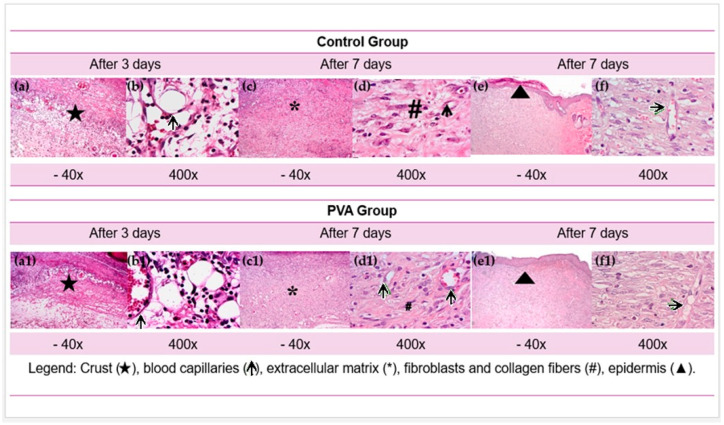
Histological aspects at experimental times of 3, 7, and 14 days of the CG and PVA groups were stained with HE (40 and 400×). Crust (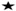), blood capillaries (**↑**), extracellular matrix (*), fibroblasts and collagen fibers (**#**), and epidermis (▲).

Figure 6 shows one of twelve images obtained by an Olympus CX31 trinocular optical microscope (model YS100) equipped with a digital camera (Bell & Howell, EU 16.0 Plus, USA). Histological images were captured at the center of each lesion. In addition, in Figure 6, the letters (a), (b), (c), (d), (e), and (f) are at a magnification of 40×; the letters (a1), (b1), (c1), (d1), (e1), and (f1) are at a magnification of 400×.

The qualitative comparative histological evaluation showed a similar epithelialization observed in both groups at the three-day experimental point, limited to the wound periphery, while the central area remained uncovered. After three days, the wounds were covered by crusts of varying thicknesses and composed of fibrin-neutrophilic material. Beneath the crusts, the wound beds were filled with young granulation tissue, marked by a highly edematous extracellular matrix (ECM) and a dense inflammatory infiltrate of neutrophils and macrophages. Numerous newly formed and congested blood capillaries were also present, indicating early stages of tissue repair.

By day seven, the edema had considerably reduced, and the ECM had become more compact. This phase showed the presence of young fibroblasts and newly formed blood capillaries. The control group (GC) continued to display focal macrophage infiltrates, with fewer freshly formed capillaries and fibroblasts than the PVA group. In contrast, in the PVA group, epithelialization had progressed to partially cover the central area of the wounds in two of the six animals, indicating a more advanced healing process.

On day fourteen, the healing process differed notably between groups. In the CG, complete epithelialization was achieved in four out of six animals, but the newly formed epidermis was immature and covered by a crust of fibrin and neutrophils. The dermis appeared edematous with focal infiltrates of macrophages and lymphocytes alongside numerous blood capillaries. In turn, all animals in the PVA group showed complete epithelialization of the injured area. The new epidermis appeared fully mature, exhibiting all layers, including the granular and cornified layers, without any crusts on the surface. The ECM in the PVA group was composed of mature, elongated fibroblasts arranged in compact, parallel bundles aligned with the epidermal surface, interspersed with sparse blood capillaries.

## 4. Discussion

Our study showed that the PVA-based electrospun dressings significantly accelerated wound healing compared to the control group. After application of the PVA dressing, macroscopic and microscopic evaluations at 3, 7, and 14 days indicated a reduction in the wound area and improved tissue regeneration. Adeli et al. [17] investigated electrospun membranes of PVA/starch/chitosan in different proportions to apply in dressings. Cell viability and cytocompatibility tests, performed with mouse fibroblasts (L929), demonstrated high viability of the membranes, ranging from 72% to 95% after 24 h and reaching 68% to 98% after 48 h, which indicates good biocompatibility. Antimicrobial assays showed efficacy against Gram-positive bacteria, such as *Staphylococcus aureus*, and Gram-negative bacteria, such as *Escherichia coli*, both associated with soft tissue infections that can interfere with the healing process. The evaluation of in vitro wound healing, performed through a scratch test based on cell migration and growth, demonstrated the efficiency of the 17 membranes in wound closure. The membrane composed of PVA/chitosan/starch, in the proportion 90/10/10, showed 100% efficacy in wound healing [21]. Both studies indicated that the treated groups presented a faster reduction in wound size compared to the control groups, highlighting the efficacy of the treatment in accelerating wound healing. These results emphasize the importance of early wound closure and the benefits of advanced formulations in promoting healing. In our study, it was observed that electrospun PVA dressings, even without associations, presented high efficiency, achieving excellent results similar to those observed in the study mentioned above, demonstrating that PVA, by itself, can be a promising option for wound treatment. Overall, the findings support the potential of polymer-based dressings to improve wound healing, offering advantages such as reduced healing time and minimal scarring.

Studies developed by Bombin, Dunne, and McCarthy (2020) highlight the effectiveness of electrospinning natural polymers. This process provides an ideal environment for wound healing, with high surface area, mesh transparency, and gas exchange, supporting cellular growth and reducing inflammation. The results from Bombin, Dunne, and McCarthy (2020) align with our findings of enhanced granulation and epithelialization [25].

The electrospun PVA dressing used in our study effectively protected the wound area due to its mesh-like structure, characterized by the empty spaces between filaments (as shown in the SEM photomicrograph), and it facilitated gas exchange, fluid movement, and exudate absorption. Combined with citric acid, these features played a key role in creating an environment that supported cell growth, reduced the risk of infection, and ultimately promoted healing. A study by Altaf et al. (2021) also highlighted the benefits of PVA-based electrospun dressings enriched with bioactive additives [26]. They found that these dressings sped up wound healing and tissue regeneration and reduced inflammation, helping wounds close faster. These findings align closely with what we observed in our study. Histopathological analysis from our research confirmed that the PVA-based dressings created optimal conditions for wound healing. This was evident through the formation of well-structured granulation tissue and accelerated re-epithelialization. Similar findings were reported by Alven et al. (2021), who observed comparable outcomes using poly(vinyl alcohol)/poly(ε-caprolactone) hybrid nanofibers [27].

PVA has a high capacity to absorb water, which facilitates the removal of exudate from wounds and reduces bacterial proliferation at the site. In addition, its properties, such as semipermeability and pH stability, are essential to create an environment favorable to cell growth [25].

Kang et al. [28] and Zhao et al. [29], investigated electrospun membranes composed of chitosan or pNSR16 associated with PVA in vivo model, relating promising results regarding wound healing time. The results showed that wounds treated with the electrospun membranes had a significantly reduced healing time compared to the control group, which received no treatment. In addition, a faster formation of granulation tissue was observed in the treated wounds, evidencing the effectiveness of these membranes in hydrating the healing process [26]. This study reinforces the idea that electrospun dressings, such as those based solely on PVA, can be a promising therapeutic approach, as collected in the preliminary results.

A study by Chang et al. (2025) showed that using PVA significantly improved the mechanical properties of the dressing, in addition to presenting biodegradable, antimicrobial, and non-toxic characteristics [24]. The tests, including an animal model and a (PVA)/GO-Cu-Cur nanocomposite, demonstrated the potential to accelerate healing, achieving 92.25% wound recovery after 14 days. These results endorse our research findings, in which we observed an equally promising performance, with 95.9% healing also in 14 days, using PVA exclusively, reinforcing the effectiveness of PVA as an effective alternative for wound treatment.

Our study’s results prove that pure PVA-CA, without nanoparticles, was highly effective in promoting wound healing. This comparison with the literature on PVA–nanoparticle composites provides valuable insights and supports the observed outcomes in our study of reduced wound area and improved tissue regeneration.

Future research can optimize PVA + CA dressings for broader clinical applications by addressing these limitations. Key limitations include scalability and cost, as the electrospinning process, while effective, may become expensive and require advanced equipment when scaled for large-scale use, potentially limiting accessibility. Patient-specific factors, such as comorbidities like diabetes, can affect wound healing rates and the dressing’s performance. Adhesion challenges are another concern, as excessive exudate or patient movement may reduce adhesion over time, necessitating additional fixation. Lastly, while the absence of nanoparticles simplifies the formulation, thorough clinical validation and regulatory approval are critical to ensure safety and efficacy across diverse patient populations.

Although our study focused on acute wound healing, the results may have relevant implications for chronic wound treatment. Providing a stable cellular matrix and reducing the inflammatory environment is crucial for acute and chronic wound healing. PVA/AC dressings, with their antioxidant and bioabsorbable properties, may help to regulate these processes, promoting a controlled inflammatory response and accelerating tissue regeneration. PVA/AC dressings may be beneficial in chronic wounds, which often present a prolonged period of inflammation and difficulties in re-epithelialization. The potential use of these dressings in combination with therapies that modulate the inflammatory response and cellular regeneration may represent an innovative approach to treating chronic wounds, such as diabetic ulcers, venous wounds, and pressure sores. In the future, additional studies on the efficacy of these dressings in chronic wound models will be essential to explore these possibilities.

## 5. Conclusions

Our research on PVA-CA electrospinning dressings for wound healing in rats presents a significant advancement by demonstrating the effectiveness of these simple, nanoparticle-free dressings in promoting wound recovery within 14 days. The results highlight that PVA–citric acid dressings improve healing outcomes and potentially enhance patients’ quality of life, regarding that chronic wounds impose substantial physiological, emotional, and economic burdens. The simplicity of the dressing’s formulation, devoid of complex nanoparticles, coupled with its cost-effective manufacturing, positions it as a practical solution for diverse healthcare contexts; this is especially relevant for settings where advanced wound care technologies may not be readily accessible. Compared to the control group, the PVA-CA-treated wounds showed superior progression in all key indicators, including granulation tissue formation, fibroblast activity, and blood vessel proliferation. The results suggest that PVA-CA dressings can be a transformative option in wound management, combining affordability, effectiveness, and ease of use. This innovation aligns with advancements in biomaterials science. It addresses a critical clinical need, paving the way for broader adoption and further research to optimize its applications in diverse medical scenarios.

## Figures and Tables

**Table 1:** Wound area size and reduction over 14 days.

Groups (*n*)		Day 0	Day 3	Day 7	Day 14
		Mean (Standard Deviation)			
W. area	Control	2.64 (0.12)	2.28(0.52)	1.86 (0.48)	0.27 (0.06)
	PVA	2.54 (0.17)	1.94 (0.28)	1.37 (0.27)	0.10 (0.07)
Statistic		*p* = 1.00	*p* = 0.99	*p* = 0.98	*p* = 0.03
%Reduc.	Control	-	−14.1 (16.8)	−29.9 (17.2)	−89.7 (2.02)
	PVA	-	−23.1 (14.4)	−45.5 (14.0)	−95.9 (2.99)
Statistic		-	*p* = 1.00	*p* = 1.00	*p* = 0.24

‘*n*’, number of samples; ‘area Size’, ‘Reduc.’, reduction (%); *p*-value from ANOVA compared post hoc.

## Data Availability

The authors declare data will be available upon request.
